# Employer Policies and Practices to Manage and Prevent Disability: Foreword to the Special Issue

**DOI:** 10.1007/s10926-016-9658-x

**Published:** 2016-08-25

**Authors:** William S. Shaw, Chris J. Main, Glenn Pransky, Michael K. Nicholas, Johannes R. Anema, Steven J. Linton, the Hopkinton Conference Working Group on Workplace Disability Prevention, Amanda E. Young, Benjamin C. Amick, Cécile R. L. Boot, Chetwyn C. H. Chan, Chris J. Main, David Gimeno, Eira Viikari-Juntura, Elyssa Besen, Fehmidah Munir, George L. Delclos, Glenn Pransky, Jean-Baptiste Fassier, Johannes R. Anema, Kelly Williams-Whitt, Kerstin Ekberg, Lois E. Tetrick, Mark G. Ehrhart, Michael Feuerstein, Michael J. Sullivan, Michael K. Nicholas, Peter Blanck, Steven J. Linton, Torill H. Tveito, Ute Bültmann, Vicki L. Kristman, William S. Shaw

**Affiliations:** 1 Liberty Mutual Research Institute for Safety, 71 Frankland Road, Hopkinton, MA 01748 USA; 2 University of Massachusetts Medical School, Worcester, MA USA; 3 Arthritis Research UK Primary Care Centre, Research Institute for Primary Care Sciences and Health Sciences, Keele University, Keele, Staffordshire, UK; 4 Pain Management Research Institute, University of Sydney at Royal North Shore Hospital, Sydney, Australia; 5 EMGO Institute for Health and Care Research, VU University Medical Center, Amsterdam, The Netherlands; 6 CHAMP, School of Law, Psychology and Social Work, Örebro University, Örebro, Sweden

**Keywords:** Employer, Disability, Disability management, Disability prevention, Research priorities

## Abstract

*Purpose* Employer policies and practices have been shown to impact workplace disability, but research in this area has waned in recent years despite an aging workforce, a growing prevalence of chronic health conditions, and a larger proportion of working-age adults on permanent work disability in many jurisdictions. The purpose of this article is to describe the background rationale and methodology for an invited conference designed to improve research of employer strategies to curtail work disability. *Methods* A multidisciplinary team of 26 international researchers with published research in employer-based disability management or related fields were invited to attend a 3-day conference in Hopkinton, Massachusetts, USA. The overall goal was to review the status of current research of workplace disability management and prevention, examine its relevance for employer decision-making, compare conceptual frameworks or theoretical perspectives, and recommend future research directions. Working groups were organized and draft manuscripts were prepared in advance. Conference activities included working group presentations and critiques, discussions with a panel of industry consultants and advisors, group interaction and debate, generation of final recommendations, and manuscript revision. *Results/Conclusion* Six principal domains were established with respect to future research: (a) further elucidation of the key workplace factors that buffer the disabling effects of injury and illness; (b) more innovative and feasible options for workplace intervention; (c) measurement of workplace-relevant disability outcomes; (d) a stronger theoretical framework for understanding the factors behind employer uptake and implementation; (e) a focus on special clinical populations and occupations where disability risk is most troubling; and (f) better representation of workers and employers that reflect the diverse and changing nature of work. Final comments and recommendations of the working groups are presented in the following six articles in this special issue of the *Journal of Occupational Rehabilitation*. Conference attendees recommended changes in methodology, collaboration strategies, and theoretical perspectives to improve the practical and scientific impact of future research of employer practices.

**Electronic supplementary material:**

The online version of this article (doi:10.1007/s10926-016-9658-x) contains supplementary material, which is available to authorized users.

Work disability represents an enormous burden for affected individuals, their employers and insurers, and for industrialized societies as a whole. With innovations in health care, increased longevity, expanded working years, and an aging workforce in many industrialized nations, the burden of work disability has never been a more pressing issue for researchers and policy makers. In the U.S., federal spending for Social Security Disability Insurance has doubled in the past 10 years [1], and 1 in 4 of American 20 year-olds can expect to be receiving SSDI disability benefits before reaching age 67 [2]. Another trend in the U.S. and elsewhere is the growing proportion of disability recipients filing for musculoskeletal conditions, mental health disorders, and other gradual-onset, chronic conditions where permanent disability might be prevented with adequate employer accommodation and support [3–7]. For these individuals, the inability to obtain or maintain employment in the wake of injury or illness can have a life-changing impact on health, family finances, and quality of life [8–11]. For those who are able to continue working, there is strong evidence that continued employment has benefits to health and well-being [12]. More research of successful employer strategies is needed to curtail work disability.

The challenge of returning injured workers to their pre-injury jobs was described in the occupational safety literature as early as 1938 [13]. According to Akabas et al. [14], “Disability management is a workplace prevention and remediation strategy that seeks to prevent disability from occurring or, lacking that, to intervene early following the onset of disability, using coordinated, cost-conscious, quality rehabilitation service that reflects an organizational commitment to continued employment of those experiencing functional work limitations” (p. 2). How to accomplish this while taking into account the contrasting viewpoints of workers, providers, employers, and insurance and disability benefit systems continues to be a vexing question for research and policy [15, 16]. Existing research suggests that employer policies and practices are critical factors in whether health symptoms or impairments will lead to a long-term work absence, job loss, or permanent disability [16–20]. Employer support includes not only discrete actions like modified duty assignments or providing assistive technology, but also more general types of support: a positive health and safety climate, non-discriminatory and inclusive leadership, social support from supervisors and co-workers, more individualized and iterative problem solving, and reasonable workplace flexibility and leeway [21–23].

Over the past 30 years, most research of disability management (DM) has focused on one of three organizational challenges for employers: (a) facilitating return-to-work (RTW) after acute onset of injury or illness; (b) enabling stay-at-work (SAW) for workers with chronic conditions or residual or recurring symptoms; or (c) providing effective accommodation and support for workers with disabilities. Common to all three are issues of accommodation, fairness, regulatory compliance, cost, coordination, tracking and surveillance, and communication. While several landmark studies in the 1980s established the cost-benefit advantage for employers to adopt proactive DM practices [13, 22, 23], employer-researcher collaborations in this area have diminished in recent years. Now, there is a need for new research to address the DM challenges of the twenty-first century: the rapidly changing nature of work in industrialized nations, the declining health of working-age adults, and the growing complexity of linkages between health care, insurance, employment, and disability systems.

On October 14–16, 2015, an invited conference of 26 researchers, representing 20 institutions in 9 countries, was convened in Hopkinton, Massachusetts, USA to evaluate the state of the science and to set a future research agenda that might reignite collaborative studies and develop and evaluate novel workplace intervention strategies to prevent long-term disability. All conference attendees had published research in the area of work disability prevention or in a related relevant field that promised new perspectives. Academic and clinical training backgrounds represented in the group included epidemiology, public health, pain management, health psychology, organizational psychology, occupational medicine, rehabilitation science, physiotherapy, occupational therapy, implementation science, law, occupational health and safety, and business management. It was a goal of the conference to strive for a trans-disciplinary approach not favoring any single dominant academic or clinical perspective.

One inherent challenge of such an international conference is the variable sets of laws, governmental systems, and cultural and societal influences affecting employer DM policies and practices across jurisdictions. There are jurisdictional differences with respect to social or private insurance systems, levels of benefit, provisions for case management and worker support, access to clinical and allied health services, worker rights and responsibilities, societal attitudes about disability and the right to work, government surveillance, and penalties for non-compliance. In some countries, disability prevention efforts of employers fall within segmented laws and private insurance programs, whereas elsewhere they are more centralized, usually through a governmental social insurance or single-payer disability benefit system. Of particular relevance are the differences in direct costs to employers for compensation of long-term sickness absence. These differences have been shown to impact return-to-work outcomes [24], but some core DM components (e.g., job accommodation, provider and worker communication, administrative processes, supervisor and co-worker support) seem fairly consistent.

Advance discussions by the conference organizing committee (the six named authors of this manuscript) identified five principal topics that were later expanded to six during the course of the conference. While not exhaustive, this list of topics was believed to cover many pertinent questions related to research methodology and relevance. The six research domains were: (a) further elucidation of the key workplace factors that buffer the disabling effects of injury and illness; (b) more innovative and feasible options for workplace intervention; (c) measurement of workplace-relevant disability outcomes; (d) advancing a theoretical framework for understanding the factors behind employer uptake and implementation; (e) a focus on special clinical populations and occupations where disability risk is most troubling; and (f) better representation of workers and employers that reflect the diverse and changing nature of work. Invited researchers were divided into small groups representing each of the topics, and draft manuscripts were produced in advance of the conference.

Working groups were instructed to summarize and analyze the existing science, to contrast this with the types of DM issues and decision-making dilemmas faced by employers, and then to generate future research recommendations. To provide a basis for this contrasting employer perspective, an initial collection of 33 employer-directed “grey literature” publications were made available to conference participants in advance of the meeting (Appendix as Electronic supplementary material). These articles were a heterogeneous collection of documents summarizing expert and legal opinions, case studies, success stories, management surveys, and best practice guidelines intended for an employer (and policy maker) audience and primarily focused on organizational efforts to manage, prevent, or accommodate disability at work. These documents were located by a web search of downloadable documents, and authors and publishers of these documents included large employers, vendors, consultants, insurers, regulatory and government authorities, employer consortiums, public policy institutes, and charitable organizations. All documents were freely available for download in English language and published in North America, Europe, or Australia/New Zealand. Because no search engine existed for a systematic and reproducible review of grey literature publications on this topic, these documents were located from an iterative keyword search of the internet using various combinations of the keyword terms “employer”, “disability”, “management”, “policy” and “guidelines”. Thus, these articles are not the product of an extensive and systematic review, but the organizing committee felt this would provide a reasonable characterization of typical policy issues and decision-making conundrums facing employers and suitable to foster discussion among researchers at the conference.

The meeting agenda included working group presentations, oral critiques and recommended revisions from other working groups, plenary discussions, and small group working sessions to edit and revise draft manuscripts. Each group was assigned to review another group’s draft manuscript in advance, and these critiques were presented and discussed as part of the conference proceedings. In addition, one afternoon of the conference included a special panel of six individuals who were known to provide regular advice and consultation to employers about optimal DM practices. Panelists described practical concerns and challenges, reacted to research recommendations in the draft manuscripts, and fielded questions from researcher participants. The purpose of the special panel was to provide real-life case illustrations of implementation and decision-making that might help to inform researcher recommendations.

Recommendations of the working groups are presented in the following six articles in this special issue of the *Journal of Occupational Rehabilitation*. Conference attendees recommended changes in methodology, collaboration strategies, and theoretical perspectives to improve the practical and scientific impact of future research of employer practices. In the first article, Kristman et al. [25] summarize workplace factors that are common to the existing literature and recommend more multi-level assessment frameworks and a broader inclusion of small and medium enterprises. In the second article, Williams-Whitt et al. [26] show the sizable gap between the types of workplace DM interventions described in randomized scientific trials and those strategies more commonly considered by employers. In the third article, Young et al. [27] examine typical workplace outcome measures assessed in DM research, and they recommend multi-level sampling in order to simultaneously address the needs of multiple stakeholders. In the fourth article, Nicholas et al. [28] explore the theories of implementation science and their potential for understanding employer uptake as part of future research protocols. In the fifth article, Pransky et al. [29] call for more DM research focusing on aging workers and those with chronic or recurrent medical conditions. In the sixth article, Ekberg et al. [30] describe the changing nature and organization of work and its implications for research of DM practices. The final article [31] provides a collective synthesis of conference proceedings and key research challenges for the future. In its entirety, this special issue provides a topical review of existing research, an analysis of strengths and limitations, gaps and opportunities for conducting practice-relevant research, and challenges for uptake and implementation. It is our hope that this collective work will help to reinvigorate work disability research that will, in turn, help assist workers to avoid unnecessary disability.

## Electronic supplementary material

Below is the link to the electronic supplementary material.

Supplementary material 1 (DOCX 16 kb)
