# Supplementary material for: Employer Policies and Practices to Manage and Prevent Disability: Foreword to the Special Issue
Source: J Occup Rehabil. 2016 Aug 25;26(4):394–8. doi: 10.1007/s10926-016-9658-x (PMC5104772; doi:10.1007/s10926-016-9658-x)
Supplement: Supplementary file 1 — Supplementary material 1 (DOCX 16 kb) [file 10926_2016_9658_MOESM1_ESM.docx]

**APPENDIX:**

**Sample “Grey Literature” publications directed to employers**

**on issues of disability management**

1. American College of Occupational and Environmental Medicine. ACOEM Guideline: Preventing needless work disability by helping people stay employed. J Occup Environ Med. 2006;48:972-987.
2. Organization for Economic Cooperation and Development. Sickness, disability and work: breaking the barriers. Paris, France:OECD Publishing; 2010.
3. Thornbory G. Back to work. Occup Health. 2005;57:14-15.
4. Watson Wyatt. Staying @Work: Effective presence at work: 2007 Survey Report Canada. Arlington, VA: Watson Wyatt Worldwide; 2007.
5. Batterson L, Fyfe BJ, Weigand D. Return-to-work programs. Business of Safety. 2010;9(2): 9-12.
6. Microlink/Global Government Forum. Disability management across the UK Civil Service: Six steps to significantly increase workforce productivity, cost effectiveness and wellbeing. Lusaka, Zambia: 2015.
7. Scott-Parker S. Moving from ad hoc to streamlined efficiency: the Lloyds Banking Group case study. London: Business Disability Forum; 2014.
8. Office of Disability Employment Policy/Job Accommodation Network. Employers’ practical guide to reasonable accommodation under the Americans with Disabilities Act. Morgantown, WV: Office of Disability Employment; 2009.
9. Towers Watson/National Business Group on Health. Pathway to health and productivity: 2011/2012 Staying@Work Survey Report. Arlington, VA: 2012.
10. Campbell Research/Heads of Workers’ Compensation Authorities. Australia & New Zealand: Return to work monitor 2011/12. Victoria, Australia: Campbell Research; 2012.
11. Hunt H. The evolution of disability management in North American workers’ compensation programs. Kalamazoo, MI: Upjohn Institute; 2009.
12. Watson Wyatt/National Business Group on Health. Dashboard for success: How best performers do it. Arlington, VA: Watson Wyatt Worldwide; 2007.
13. Institute for Work & Health. Seven ‘principles’ for successful return to work. Toronto, Ontario: Institute for Work & Health; 2014.
14. Adya, M., Cirka, C., & Mitchell, K. Final Report: Corporate Return to Work Policies and Practices: A National Study. Syracuse, NY: Burton Blatt Institute, Syracuse University; 2012.
15. European Agency for Safety and Health at Work. Work-related musculoskeletal disorders: Back to work report. Brussels, Belgium: European Agency for Safety and Health at Work; 2007.
16. International Labour Office. Disability in the workplace: employers’ organizations and business networks. Geneva, Switzerland: International Labour Office; 2011.
17. International Labour Office. Managing disability in the workplace. Geneva, Switzerland: International Labour Office; 2002.
18. CIGNA Corporation. Employer’s guide to creating a successful return-to-work (RTW) program. Hartford, CT: CIGNA Corporation; 2009.
19. Workers’ Compensation Board of Nova Scotia. Return-to-work: Getting started. Halifax, Nova Scotia: Workers’ Compensation Board; 2015.
20. Bunn W, Baver R, Ehni T, Stowers A, Taylor D, Holloway A et al. Impact of a musculoskeletal disability management program on medical costs and productivity in a large manufacturing company. Am J Manag Care. 2006;12:27-32.
21. Morrison KW. Returning to work, successful programs can help lower employer costs, including workers’ compensation. Safety + Health, Official Magazine of the NSC Congress & Expo; Oct 2014.
22. European Foundation for the Improvement of Living and Working Conditions. Employment and disability: Back to work strategies. Loughlinstown, County Dublin, Ireland: European Foundation for the Improvement of Living and Working Conditions; 2004.
23. International Social Security Association. Who returns to work and why? Evidence and policy implications from a new disability and work reintegration study. Geneva, Switzerland: International Social Security Association Research Programme; 2002.
24. Wlekinski B, Salon R, Taylor B. Best practices in employee retention and return-to-work: an in-depth look inside an exemplary American corporation. Washington, D.C.: Office of Disability Employment Policy; 2014.
25. Towers Watson/National Business Group on Health. The Health and Productivity Advantage: 2009/2010 Staying@Work Report. Arlington, VA: Towers Watson; 2012.
26. Mitchell K. The return to work dividend: Protecting productivity (Testimony to the US Senate Committee on Health, Education, Labor and Pensions). Washington, D.C.: Kenneth Mitchell; 2012.
27. International Labour Office. Code of practice on managing disability in the workplace. Geneva, Switzerland: International Labour Office; 2001.
28. Disability Management Employers Coalition. Best practices in return to work. San Diego, CA: Disability Management Employers Coalition; 2011.
29. PricewaterhouseCoopers. The future of work, a journey to 2022. New York, NY: PricewaterhouseCoopers; 2014.
30. Manyika J, Lund S, Auguste B, Ramaswamy S. Help wanted: The future of work in advanced economies. San Francisco, CA: McKinsey Global Institute; 2012.
31. Chan C, Morgan J, & Pfeiffer J. The future of work, reshaping the workplace today: Building for tomorrow. San Francisco: Chess Media Group; 2011.
32. Main J, Phillips C, Farr A, Thomas A. Health and work: minimising the problems of musculoskeletal pain. Manchester, UK: Wales Health Work Partnership; 2004.
33. Bloom DE, Cafiero ET, Jané-Lopis E, Abrahams-Gessel S, Bloom LR, Fathima S et al. The global economic burden of noncommunicable diseases. Geneva: Global Economic Forum; 2011.
